# Effective Coverage: A Metric for Monitoring Universal Health Coverage

**DOI:** 10.1371/journal.pmed.1001730

**Published:** 2014-09-22

**Authors:** Marie Ng, Nancy Fullman, Joseph L. Dieleman, Abraham D. Flaxman, Christopher J. L. Murray, Stephen S. Lim

**Affiliations:** Institute for Health Metrics and Evaluation (IHME), University of Washington, Seattle, Washington, United States of America

## Abstract

A major challenge in monitoring universal health coverage (UHC) is identifying an indicator that can adequately capture the multiple components underlying the UHC initiative. Effective coverage, which unites individual and intervention characteristics into a single metric, offers a direct and flexible means to measure health system performance at different levels. We view effective coverage as a relevant and actionable metric for tracking progress towards achieving UHC. In this paper, we review the concept of effective coverage and delineate the three components of the metric — need, use, and quality — using several examples. Further, we explain how the metric can be used for monitoring interventions at both local and global levels. We also discuss the ways that current health information systems can support generating estimates of effective coverage. We conclude by recognizing some of the challenges associated with producing estimates of effective coverage. Despite these challenges, effective coverage is a powerful metric that can provide a more nuanced understanding of whether, and how well, a health system is delivering services to its populations.

## Abstract 2

As part of the PLOS Collection on Universal Health Coverage, Stephen Lim and colleagues review the concept of effective coverage and discuss the ways in which current health information systems can support generating estimates of effective coverage.

This paper is part of the PLOS Universal Health Coverage Collection.

**Summary Points**

- Effective coverage unites intervention need, use, and quality into a simple but data-rich metric, reflecting the core components of UHC.
- Effective coverage can be applied to understand the health gains delivered by interventions at a range of levels, from individual benefits to national impact.
- Effective coverage can be measured and used across resource settings. Lower-income countries can harness data from existing survey data to feed into effective coverage estimations.
- The broader use of effective coverage remains hindered by the availability and quality of health data, especially at subnational levels.

## Introduction

Strengthening health systems, ensuring affordability of care, improving access to quality services, and building capacity are core tenets of universal health coverage (UHC). In 2010, the World Health Organization (WHO) called for concerted efforts to achieve UHC, with reducing disparities and promoting opportunities for obtaining quality care with financial protection as WHO's underlying goals [1]. The ideology of UHC laid out by WHO was viewed as ambitious and noble; however, it has been criticized for the lack of specificity for defining milestones crucial for monitoring progress [2]. The importance of systematically tracking the progress in attaining UHC was highlighted in the 2013 World Health Report [3], which drew attention to the dearth of empirical evidence for assessing and informing policies related to UHC. The report identified several key research priorities, which included deepening the understanding of disease burden at the country level and identifying policy-relevant metrics for tracking progress. Nevertheless, the question remains: how should progress towards UHC be monitored?

As noted in a recent World Bank report on UHC [4], in order to adequately capture the spectrum of health services represented by UHC, “a more holistic approach to the dimensions of access needs to be understood.” In other words, the most useful metric for monitoring progress in UHC should encompass the multifaceted nature of UHC. The monitoring framework put forth by WHO and the World Bank Group in 2013 highlighted two major components critical to assessing UHC progress, namely, service coverage and financial protection coverage for all people [5]. For measuring service coverage, the concept of effective coverage was noted. In contrast to crude coverage, which focuses solely on intervention access or use, effective coverage is a measure that unites intervention need, use, and quality. The comprehensiveness of this metric makes it more suitable for monitoring UHC [6]–[9]. In this paper, we will review the concept of effective coverage and discuss a number of key issues related to its measurement.

## What Is Effective Coverage?

Effective coverage is defined as the fraction of potential health gain that is actually delivered to the population through the health system, given its capacity. It is comprised of three components, namely, need, use, and quality. *Need* refers to the individual/population in need of a particular service; *use* refers to the use of services; and *quality* refers to the actual health benefit experienced from the service. Measuring effective coverage is a significant advancement over the usual approach of measuring crude coverage, which only captures access conditional on need. In particular, given that use of service alone does not imply that the full benefit of the service is being realized, it is crucial for a health performance metric to capture not only coverage but also quality. The calculation of effective coverage is summarized in Box 1.

**Box 1. Formal Definition of Effective Coverage**

At the individual level, effective coverage is defined as the fraction of potential health gain that is actually delivered to the population through the health system, given its capacity. The formal definition is as follows:
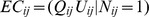
where *EC_ij_* is the effective coverage of individual 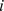 with intervention *j*; *Q_ij_* is the expected quality of intervention *j* as delivered to person *i*; *U_ij_* is the probability of individual *i* receiving intervention *j*; and *N_ij_* is an indicator of whether individual *i* is in need of intervention *j*.

At the population level, effective coverage for a given intervention is an aggregate of each individual's probability of effective coverage. To estimate effective coverage for a specific intervention *j* at the population level, individual-level effective coverage is aggregated as follows:
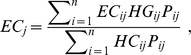
where *HG_ij_* is the expected health gain from the intervention and *P_ij_* is the probability of an individual needing the intervention.

To estimate overall effective coverage for the health system of a country, the effective coverage for a set of interventions is further aggregated as follows:
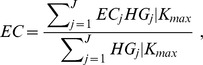
where actual expected health gain is conditioned on the maximum performance *K_max_*.

In addition to capturing quality, effective coverage has another unique strength: it is a very flexible metric that can easily be adapted for different contexts and assessed at different administrative levels. Specifically, effective coverage can be measured for one single intervention and provide information on specific intervention roll-out. To the degree that data are available, effective coverage can also be aggregated across a large, diverse set of interventions and proxy the effectiveness of an entire health system. In other words, effective coverage can be adapted in a manner that reflects country needs and health priorities, hence serving as an appropriate indicator for tracking progress and benchmarking performance. Effective coverage also can be estimated for subnational levels or population subgroups, which helps to pinpoint the geographic areas or populations lagging behind in the receipt of effective interventions. Obtaining subnational-level estimates is particularly important when effective coverage is used to monitor progress towards UHC, as UHC has a noteworthy equity agenda and subnational estimates are of the utmost importance.

**Figure 1:**
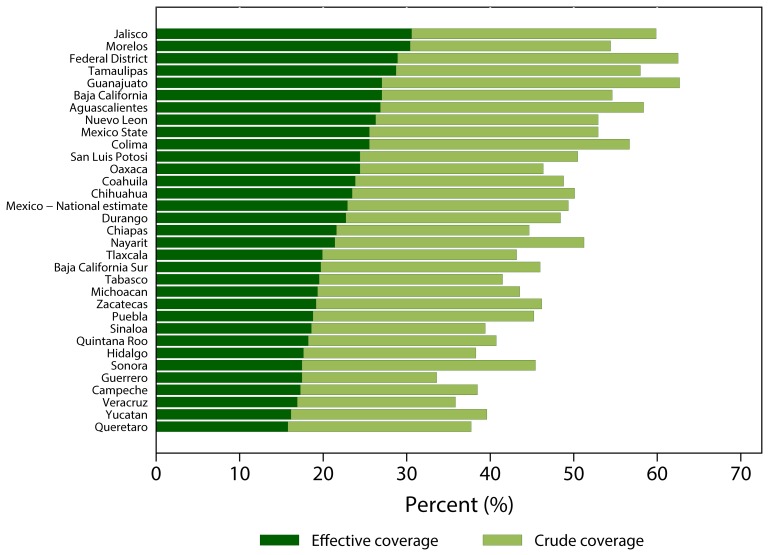
Crude and effective coverage of hypertension treatment across Mexican states, 2005–2006.

To illustrate how effective coverage has been applied in the real world, the experience of Mexico is one of the most comprehensive examples. In an effort to benchmark progress towards improved health services and evaluate the impact of its country-wide health reforms, the Mexico Ministry of Health adopted effective coverage metrics in 2001. A set of interventions reflective of the priority health needs were included in the metric, and effective coverage estimates were derived for each state [10]. The results enable the Ministry to pinpoint gaps in intervention access *and* places where intervention effectiveness was sub-optimal. Figure 1 shows how effective coverage can reveal gaps in access and inadequacy in service delivery. Specifically, the discrepancy in crude and effective coverage of hypertension treatment across states shows that in some areas the intervention delivered might not have achieved the desired health outcome despite a high level of access.

The estimation of effective coverage, however, can be challenging because some of the key components can be difficult to measure directly and data quality can vary. In the following section, we discuss factors that can affect the collection and use of effective coverage in applied settings.

## Practical Considerations in Applying Effective Coverage

In order to optimally use effective coverage as a metric for monitoring healthy system improvement or progress towards UHC, a country has to consider several factors. First, a country must identify its overall health needs and priorities. Second, a country has to develop specific strategies for collecting data on need, use, and quality of selected interventions. Third, a country has to devote resources to enhance both national and subnational capacity to collect and monitor health information.

### Identify Priorities for Effective Coverage Indicators

Given the broad range of health services delivered by health systems today, measuring effective coverage for every intervention would be impossible. When effective coverage was used to benchmark state-level health system performance in Mexico [10], the Ministry of Health selected a subset of interventions that most directly aligned with the country's health priorities. These selected interventions included a mixture of maternal and child health interventions (such as immunizations, antenatal care, and skilled birth attendance) and interventions for non-communicable diseases (such as cancer screening and hypertension treatment). While these selected interventions were far from exhaustive, they appropriately reflected Mexico's health needs and priorities throughout the country.

Health need serves as one of the guiding principles in determining what should be prioritized and included for estimating and tracking effective coverage. Individual countries' health needs are likely to vary, but some similarities can be found across income levels, geographic location, and cultures. The Global Burden of Disease 2010 (GBD 2010) study helped to illuminate some of these trends in health needs [11]. For instance, among higher income countries, non-communicable diseases compose most of their health burdens and corresponding needs. Countries that have transitioned from lower to higher levels of income often experience a parallel transition in health needs, largely shifting from disease burdens caused by communicable diseases to those caused by non-communicable conditions. Lower-income countries still experience the largest health burdens from infectious diseases and maternal and child conditions, but many of them have documented gradually rising rates of injury and more chronic ailments.

This diversity of disease burdens across countries implies that what comprises UHC is likely to vary across settings. For instance, among lower-income countries, UHC may focus around achieving basic healthcare for all populations and prioritizing access to interventions that address infectious diseases and maternal and child health conditions [12]. Therefore, the interventions included in estimating effective coverage would align with these health need priorities, such as antenatal care, skilled birth attendance, and critical surgical procedures [13]. For higher-income countries, UHC is likely to primarily focus on improved access to treatment of and preventive services for non-communicable diseases [14]. Subsequently, the interventions included for effective coverage estimation for these settings would need to be related to managing chronic conditions. Data visualization tools can help identify country-specific disease burdens and health needs: http://www.healthdata.org/results/data-visualizations.

Considerable differences exist across and within world regions, but a number of commonly experienced disease and injury burdens exist among subsets of countries. For instance, national disease burden studies conducted in the United States, the United Kingdom, and China identify ischemic heart disease, chronic obstructive pulmonary disease (COPD), stroke, and lung cancer as the leading causes of premature mortality and disability for all three countries [15]–[17]. This finding implies that some diseases could be treated as regional, and potentially global, health needs for monitoring effective coverage.

Identifying a country's health needs and corresponding interventions to address them is a necessary consideration, but it is not sufficient. It is also critical to consider the cost-effectiveness and sustainability of a given intervention or set of interventions within the health system delivering them. The interventions selected for tracking effective coverage should align with country-specific health needs *and* a country's financial and administrative capacity to support their provision over time. Efforts have been made to compile data on optimal intervention delivery options. This body of work includes WHO's Choosing Interventions that are Cost Effective (WHO-CHOICE) [18] and the ongoing projects within the Disease Control Priorities framework [19]. Through these projects, information on intervention costs and effectiveness has been generated and then assembled such that comparisons can easily be made across intervention packages and delivery options. However, these kinds of data are generally only available at more macro-levels, and have yet to be systematically produced at the country level.

When defining key interventions to track for effective coverage, it is also important to recognize that priorities of health care and intervention are not solely driven by the disease burden and cost-effectiveness. Other considerations, including equity, fairness, individual rights, and historic and cultural concerns, all play a significant role in shaping health system and measurement agendas [20]. For instance, some health interventions may be widely delivered to a population to address social demands and inequity — even if these interventions do not align well with the population's health needs or the knowledge base of intervention cost-effectiveness [21].

### Tracking Intervention Need, Use, and Quality

To estimate effective coverage, the metric's three components — intervention need, use, and quality — need to be measured in a consistent way. Data on a person's need for an intervention, use or exposure to an intervention, and if the intervention had its intended effect (usually measured by a biological marker or health outcome) have to follow an information continuum, such that these three factors can be linked together.

Here we define the measure of intervention need as whether an individual would benefit from receiving a specific health intervention. Intervention use reflects whether an individual, conditional on needing the intervention, received or used a specific intervention. Intervention quality captures whether a specific intervention actually conferred the health gain or protection it was supposed to (effectiveness). To measure effective coverage of diabetes management, for instance, information would need to be collected on (1) the prevalence of diabetes in a population (i.e., individuals who need treatment for diabetes); (2) the proportion of people with diabetes who receive treatment; and (3) the effectiveness of their treatments (i.e., whether levels of fasting plasma glucose declined with treatment) [22].

In the following sections, we will focus on discussing the approaches and challenges for measuring each of these components.

## Measuring Intervention Need

Intervention need can be defined in different ways. First, intervention need can be viewed in normative terms. For instance, pregnant women would be considered the population in need of antenatal care, and children younger than one year old would be the population in need of the pentavalent vaccine. In places with well-developed health information systems, the number of pregnant women and young children in a population can be tracked with relative ease. However, in settings with less developed health information systems, this information may not be as easily attainable. Nationally representative surveys, such as the multi-country Demographic and Health Surveys (DHS), serve as efficient mechanisms for obtaining population information in places with less robust information systems. In these surveys, retrospective information on pregnancy and child births are routinely collected, which can offer insights into recent needs and demand for relevant health services. However, these surveys do not routinely capture all health needs that a population might experience. The DHS, for example, primarily collects data on communicable disease and maternal and child health conditions, largely excluding the measurement of non-communicable diseases, mental health, and injury. As a result, routinely measuring health needs within these health domains is likely to require alternative strategies.

Second, intervention need can be determined by diagnosis, which allows for the targeting of specific populations for interventions. For instance, individuals with HIV/AIDS need antiretroviral therapy (ART) when they meet specific CD4 count thresholds. Individuals who meet diagnostic criteria for depression, as set forth by the latest version of the Diagnostic and Statistical Manual of Mental Disorders (DSM), would be the population in need of antidepressants, therapy, or a combination of treatments. National surveys often collect information on self-reported health state, which can provide some insights into population-level health needs. However, many types of health conditions may be less reliably captured through self-report or may be prone reporting biases (e.g., estimating one's weight and height or self-reported HIV status) [23]. Using biological markers provides more accurate measures of various health conditions [24], and many national surveys regularly collect data on biological markers throughout the world. Examples include the National Health and Nutrition Examination in the United States, China's Chronic Disease Risk Factor Surveillance, and the DHS's routine collection of hemoglobin samples to test for anemia. In some countries, biological markers for sexually transmitted diseases (STDs) and HIV also are collected [25].

Biological data collection can provide more objective measurements of health needs, but it is important to consider diagnostic thresholds and limitations. There has been substantial debate over the appropriate CD4 count thresholds for ART initiation [26], and similar disagreements have occurred over threshold definitions for conditions such as hypercholesterolemia and hyperglycemia [27]. This lack of consensus over threshold standards means that estimating health needs can be highly influenced by whatever threshold is selected by clinicians and researchers.

In addition to surveys, surveillance data collected through clinics and hospitals serve as another major source of health need data. For instance, population-level estimates of HIV prevalence are often derived from routine blood tests conducted at antenatal clinics. These facility-based databases serve as convenient sources, but they may not provide fully representative information on broader population needs. Health facility records are unlikely to capture the health needs of individuals who do not regularly seek health services (or the services provided by specific types of facilities, such as antenatal clinics), so these sources of data tend to under-represent the least wealthy populations [28].

Recent progress in verbal autopsy (VA) methods provides alternative tools for measuring health needs in resource-constrained settings. For example, the Symptomatic Diagnosis (SD) approach generates a probabilistic “diagnosis” for conditions by using self-reported symptoms collected during interviews. This approach can supplement data collection when collecting biological markers or providing clinical assessments are not feasible. An SD pilot in Mexico has sought to identify cases of several non-communicable diseases, including angina asthma, chronic obstructive pulmonary disease, vision loss, hearing loss, depression, and osteoarthritis [26]. Preliminary results indicate that SD outperforms current questionnaire-based epidemiological approaches in diagnosing diseases such as depression, angina, and asthma [29]. The chance-corrected concordance was above 75% and as high as 93%. Further, the absolute error associated with SD applications was up to four times lower than current methods for some conditions.

## Measuring Intervention Use Conditional on Need

The use of an intervention is a central component of estimating effective coverage. Specifically, it is defined conditional on need. In other words, it is essential to measure not only the number of individuals using a service, but also differentiate the number of individuals *in need* who are using it. When need is defined normatively, intervention use among those in need may be more easily measured by the total number of individuals belonging to a particular demographic category who have accessed an intervention, for example the number of pregnant women who have attended antenatal care (ANC) or the number of children younger than 12 months old who have received the pentavalent vaccine. Properly measuring intervention use or exposure and tracking intervention coverage over time can be challenging, especially because intervention data sources are often subject to inconsistencies and information gaps [30].

Data on intervention use can be extracted from several sources, including administrative systems and household surveys. Administrative health databases generally offer the most complete records of intervention use over time (e.g., the number of insecticide-treated nets [ITNs] distributed each year), which is immensely helpful for computing trends in intervention coverage. At the same time, administrative sources often experience a variety of reporting biases and may not link the receipt of an intervention to an individual's need for it. Household surveys generally provide more robust estimates of intervention coverage, but the gaps in time between survey administrations can make tracking intervention trends difficult. As a result, many studies and programs have triangulated data or combined estimates of intervention use from multiple sources through statistical modeling [31].

Different strategies for data validation and synthesis are regularly used to estimate trends in intervention coverage. For example, expert groups have assessed the most appropriate analytical methods for WHO and UNICEF to use in estimating immunization intervention coverage [32]. In other cases, systematically testing different modeling strategies has been the predominant approach. For example, a Bayesian model applied a systems dynamic framework to bring together multiple sources of data on ITNs, ranging from ITN delivery records from manufacturers to household survey measures of ITN ownership, to construct annual estimates of ITN coverage [33]–[34]. This modeling approach demonstrated how capturing multiple measures along a distribution chain can support the annual estimation of intervention coverage.

## Measuring Intervention Quality

Capturing whether the intended health benefit was provided by an intervention is what differentiates effective coverage from more traditional “crude” intervention coverage. This requirement for additional information, which is generally measured by a biological marker or observable health outcome, makes effective coverage more challenging to assess than coverage alone. Although assessing intervention quality is often the most complicated aspect of estimating effective coverage, several approaches have been proposed and tested under routine settings [35]–[36]. These approaches include content of care, biomarkers, cohort registration, exposure matching, statistical methods, and risk-adjusted outcomes. Table 1 gives an overview of the strengths and limitations of the approaches, which all provide a quantitative means to estimating the health gain associated with the receipt or use of a specific intervention.

**Table 1:** Approaches to measuring effective coverage.

Approach	Description	Study examples	Potential data sources	Strengths	Limitations
Content of care	- Focuses on the health care process - Involves indicators that target the resource and activity outputs of an intervention	- WHO Quality assessment and assurance in primary health care [37]	- Hospital databases- Patient exit interviews	- Offers information from both demand- and supply-side factors- Resource and activity outputs can serve as objective indicators	- Subjectivity in patient assessments of quality- High outputs or content of care may not directly translate into health gains
Biomarkers	- Focuses on the health benefits that can be detected biologically	- Assessment of vaccine effectiveness [39]	- Health surveys that include physical examinations	- Provides an objective measure of actual health gains or impact	- Collection of biomarker data can be costly and not always feasible in resource-constrained settings- Not applicable to all health conditions
Cohort registration	- Focuses on changes in individual health outcomes over the course of treatment	- Assessment of highly active antiretroviral therapy (HAART) [41]	- Cohort registration databases	- Provides measurement of treatment effectiveness for chronic conditions over time	- Limited to interventions that involve close patient monitoring and treatment by healthcare providers- Requires careful consideration of time-dependent confounding factors and lost to follow-up
Exposure matching	- Compares health outcomes of individuals who had intervention exposure to those who did not have exposure to an intervention	- Assessment of health impact of IPTp and ITNs [43]	- Household survey data	- Allows for the quantification of the health gains associated with intervention exposure by calculating odds ratios or relative risks with existing data	- Household surveys are rarely powered to detect health effects- Unmeasured confounding factors need to be accounted for due to the observational nature of analysis
Statistical methods	- Uses statistical and econometric techniques, such as instrumental variables (IVs) and matching, to estimate health outcomes while controlling for unobserved variables	- Assessment of diabetes and hypertension management in Iran [45]	- Health survey data	- Offers a convenient solution to address potential biases associated with confounding factors	- Only approximates the relationship, or correlation, between intervention exposure and a health outcome rather than the causal effect
Risk-adjusted outcomes	- Estimates health outcomes while accounting for the patient characteristics and risks of death that can vary systematically across sites	- Birth weight–adjusted neonatal mortality [46]	- Hospital databases	- Provides an indicator for quality of care that reflects both procedural outputs and the health impact of received care	- Limited to interventions that are delivered at health facilities- Certain risks may not be easily adjusted for if they are challenging to quantify

### Content of Care

Content of care focuses on the health care process. It often involves indicators that target the resource and activity outputs of an intervention [37]. Information on these indicators can be obtained through both providers and beneficiaries. Asking respondents about the frequency, timing, and content of antenatal care in household surveys, including the type of provider and type of tests accompanying ANC visits, is an example of a beneficiary-based approach. However, one of the major limitations of using content of care as a measure of quality is that high content of care does not always associate with positive health gain. This discrepancy is demonstrated in a recent study on patient satisfaction and health outcomes conducted in the United States [38]. Using national survey data, the study found that high patient satisfaction was not associated with positive health outcome but rather higher mortality.

### Biomarkers

One way to objectively measure quality in terms of the actual health benefit of an intervention is to evaluate biomarkers. This approach was used in a study comparing the effectiveness of a one- versus two-dose regimen of measles-mumps-rubella (MMR) vaccine [36]. Single-dose MMR vaccine is common in many countries. Although clinical studies have demonstrated poor efficacy for single-dose MMR, the effectiveness (or the lack thereof) of the regimen at the population level has not been investigated empirically. By evaluating the biomarker levels, specifically MMR IgM and IgG antibody, of over 1,000 children who had received one- versus two-dose of MMR, researchers showed that measles and mumps IgG antibody levels were considerably lower than putative levels among children who had only one dose of the vaccine. This study provided empirical evidence in support of reinforcing a two-dose vaccine regimen in order to achieve population-level immunity [39]. However, obtaining biomarker measures is not always feasible due to resource constraints as well as the nature of diseases.

### Cohort Registration

For interventions that involve close patient monitoring and treatment by care providers, the most fitting approach for tracking effectiveness is cohort registration. One example is the WHO's strategy for treating tuberculosis, known as DOTS (directly observed treatment, short-course). Through the program, new cases of tuberculosis are recorded; adherence and treatment outcomes are also documented. By evaluating individuals' information over time, one can obtain direct quality measure of the effectiveness of intervention. When analyzing cohort data, time-dependent confounding factors must be taken into consideration. A good example of this approach is the study of highly active antiretroviral therapy (HAART) by Sterne et al [40]. By controlling for time-dependent covariates including CD4 counts and HIV-1 RNA concentration, it was found that HAART substantially reduced the rate of progression to AIDS or deaths by 86%. Another issue that requires proper attention when analyzing cohort data is when patients are lost to follow-up. A recent study examining the effect of lost to follow-up among HIV-infected individuals showed that estimated survival with and without adjustment of mortality amongst those lost to follow-up can lead to overestimation of the treatment effect by as much as 40% [41]. Despite the rich information contained in cohort registration, maintenance of the system can be costly.

### Exposure Matching

Effectiveness of an intervention can also be inferred using household survey data by comparing the health outcomes of individuals with and without exposure to the intervention. This strategy is known as exposure matching. Exposure matching has been applied in a variety of contexts, including the assessment of the effectiveness of malaria control interventions. Using household survey data, a recent study compared birth weight and survival of infants whose mothers had had different exposures to ITN and/or intermittent preventive therapy during pregnancy (IPTp). The study found that exposure to full malaria prevention with IPTp or ITNs reduced the risk of neonatal mortality and the odds of low birth weight by 18% and 21%, respectively [42].

It is important to note, however, that household surveys are often not powered to detect health effects in this way. As a result, although aggregate level estimates of intervention effects across countries are detectable, variation across countries is less easily measured. To facilitate the measurement of intervention effectiveness at a finer scale, increasing in sample size for household surveys on a periodic basis can be considered. Another important caveat when carrying out exposure matching using survey data is the presence of unmeasured confounding factors as the analyses are observational retrospective in nature.

### Statistical Methods

As illustrated in the previous sections, because of the presence of potential confounding factors, it is difficult to immediately attribute a health outcome to the effectiveness of an intervention in retrospective or cross-sectional analyses. One way to tackle this limitation is to apply statistical and econometric methodologies, such as instrumental variable (IV) and matching. IV was used in a recent study assessing the effectiveness of influenza vaccination among elderlies in Ontario, Canada [43]. Contrary to previous research, the study found no significant association between influenza vaccination and all-cause mortality. In another study, the effectiveness of diabetes and hypertension management intervention in Iran using mixed-effect models and propensity score matching [22]. The results suggested an association between the intensity of primary diabetes and hypertension management intervention and improved health outcomes. Although these statistical techniques serve as convenient solutions to the issue of confounding factors, they only approximate the correlation, rather than causation, between intervention and effect.

### Risk-Adjusted Outcomes

For intervention delivered in hospital settings, one approach to capture effectiveness is to estimate the risk-adjusted outcomes. Risk-adjusted outcomes such as risk-adjusted hospital mortality and risk-adjusted 29-day mortality serve as proxies for the quality of hospital care while taking into account the fact that the patient characteristics and risks of death may vary systematically across sites. In a recent study by Straney et al [44], the variation in neonatal clinical care across the US was investigated. By examining the gestational age and birth weight-adjusted (GA-BW) neonatal mortality from 1960–2006, the study concluded that the quality of obstetrical and neonatal care have indisputably improved over time. One of the limitations of risk-adjusted outcomes, however, is that certain risks may not be easily quantifiable and hence adjusted for.

It is important to emphasize that the notion of quality in effective coverage not only reflects the content pertaining to health services, which measures the resource and activity outputs of an intervention [37]. More importantly, it directly captures intervention impact on health under routine conditions. Capturing health impact in the quality component is critical because high levels of content do not necessarily translate into optimal health outcomes and impact. Effective coverage measured in this manner can help more precisely pinpoint gaps in health service delivery, especially if levels of effective coverage are compared with crude measures of intervention coverage. For example, if two districts have similar crude coverage of 80% for an intervention, but one has 75% effective coverage and the other has 50% effective coverage of the same intervention, this finding would imply differential delivery and quality of care. Thus, the policy recommendation for the two districts will vary dramatically. Effective coverage is designed to be a flexible and powerful health metric that can uniquely help to understand actual health system performance.

## Building Capacity for Tracking Effective Coverage

Having both the infrastructure and human resources needed to optimally track effective coverage is critical to the metric's application. In many settings, one of the major obstacles is a lack of reliable data. Strengthening health information system capacity should occur alongside the implementation of health reforms for achieving UHC. Ideally, health information systems would capture both supply- and demand-side data on health services, and then support the triangulation of data. The need for further strengthening of health information systems is not limited to lower-resource settings; it is a challenge experienced by higher- and lower-income countries alike [45]–[47].For instance, a United States–based study recently created a population-based health surveillance system for tracking disparities in chronic diseases across ethnic groups in King County, Washington [48]. This system sought to integrate multiple sources of administrative data, including medical discharge records, reportable conditions, payer data, and Medicare files, with data collected from surveys and physical examinations. Through these triangulation efforts, King County had a more accurate mechanism for tracking the health needs of its entire population, relying less on individual contact with the health system. A similar integrative health information system is under development in the Kingdom of Saudi Arabia [49].

Because health policies are often being driven and executed at a local-level, the ideal integrated health information system should not only be designed to enable estimations of effective coverage at the national level, it should also allow estimations of the metric at the subnational level [16],[50]–[53]. Derivations of effective coverage at subnational level have been challenging because of the lack of dependable and representative data sources. Limitations in human resources and technical supports hinder regular collection of health data at the local level. Moreover, many surveys implemented sampling schemes that are designed to be representative at the national level only. In some cases, post-stratification weighing strategy can be applied. However, that does not guarantee that the estimates accurately represent the actual local demographic composition. Advancement in small area methodologies, which capitalized on geographical relatedness, has substantially enhanced our capacity to maximize the use of existing data to estimate and evaluate disease prevalence and intervention coverage at the local level [54]. Nevertheless, a solid data collection platform is crucial for long-term monitoring and policy planning.

## Conclusions

UHC entails a global health ideal which, with concerted effort, could become reality. At the same time that policymakers and health officials are developing strategic plans for achieving UHC for their countries, it is also critical to prioritize establishing a data-driven framework for tracking progress in achieving UHC. In doing this, governments foster responsiveness and accountability for their UHC aims.

In this paper, we have reviewed the concept of effective coverage and highlighted three main components that affect its use under routine conditions. Box 2 provides recommendations on the major considerations for tracking effective coverage. These include first, reviewing existing evidence on disease burden, affordable interventions and social priorities; second, developing strategies to measure needs, use, and quality; and third, building system capacity for continuous monitoring. Among these considerations, building capacity for data collection and use remains the most substantial hurdle in broadly using effective coverage. Without further developing the strength and representation of routine health information systems, tracking national and subnational progress towards health goals, such as UHC, is likely to be more resource-intensive and prone to suboptimal accuracy.

**Box 2. Recommendations**

1. **Identify disease burden, affordable interventions, and social priorities when selecting which interventions to include in estimating effective coverage**
  - Examine existing evidence on health need priorities.
  - Evaluate cost-effectiveness of interventions.
  - Consider concerns of equity: political, social, and cultural
2. **Develop measurement strategies for tracking need, use, and quality for selected interventions**
  - Intervention need can be measured using existing survey data, biological markers, or alternative methods such as Symptomatic Diagnosis (SD).
  - Intervention use can be estimated by synthesizing administrative and household survey data.
  - Intervention quality can be determined by different methods that link health outcomes with the receipt or use of interventions. Examples of these approaches include exposure matching, risk-adjusted health outcomes, and statistical modeling.
3. **Build capacity for measuring effective coverage**
  - Develop an integrated health surveillance system that allows triangulation of data.
  - Devote resources for training staff on data collection and basic analysis.

As health reform efforts continue to evolve worldwide, the range and scope of interventions comprising UHC priorities are likely to change over time as well. By harnessing existing health information systems and expanding their capacity, countries can be in the position of using effective coverage to align with their own UHC needs and to more accurately monitor progress towards their UHC goals.

## Abbreviations

ANC: antenatal care
ART: antiretroviral therapy
COPD: chronic obstructive pulmonary disease
DHS: Demographic and Health Survey
DOTS: directly observed treatment, short-course
DSM: Diagnostic and Statistical Manual for Mental Disorders
GA-BW: gestational age and birth weight–adjusted
GBD 2010: Global Burden of Disease 2010 study
HAART: highly active antiretroviral therapy
IPTp: intermittent preventive therapy during pregnancy
ITN: insecticide-treated net
IV: instrumental variable
MMR: measles-mumps-rubella
SD: Symptomatic Diagnosis
STDs: sexually transmitted diseases
UHC: universal health coverage
VA: verbal autopsy
WHO: World Health Organization
